# Advancing the frontier of rare disease modeling: a critical appraisal of in silico technologies

**DOI:** 10.1038/s41746-025-02068-1

**Published:** 2025-11-17

**Authors:** Francesca Pistollato, Fabia Furtmann, Lindsay J. Marshall, Surat Parvatam, Jan Turner, Flora Tshinanu Musuamba, Giulia Russo, Francesco Pappalardo

**Affiliations:** 1 Humane World For Animals, Brussels, Belgium; 2 Humane World For Animals, Washington, D.C, NW USA; 3 Humane World For Animals, Hyderabad, India; 4 University of Namur, NAmur Research Institute for LIfe Sciences (NARILIS), Clinical Pharmacology and Toxicology Research Unit, Namur, Belgium; 5 Federal Agency for Medicines and Health Products, Brussels, Belgium; 6 Department of Drug and Health Sciences, The COMBINE Group, University of Catania, Catania, Italy

**Keywords:** Diseases, Computational models

## Abstract

Rare diseases affect over 300 million people worldwide and pose unique research challenges. In silico approaches, such as mechanistic models, machine learning, and simulations, offer scalable tools for disease characterisation, drug discovery, and virtual trials. This review categorises these methods by context of use, critically appraises their strengths and limitations, and identifies barriers to translation, highlighting key opportunities and ongoing challenges in advancing computational strategies for rare disease research.

## Introduction

Rare diseases, defined by low prevalence, collectively affect over 300 million people worldwide, representing a major public health and research challenge^1^. These conditions are often characterised by unclear etiology, variability in genotype-phenotype relationships, and highly heterogeneous clinical trajectories. Compounding these difficulties are small patient populations, limited access to biological samples, and a lack of validated biomarkers or endpoints, all of which hinder traditional research and therapeutic development efforts^2^.

Historically, rare disease research has relied heavily on animal models, which are often ill- suited to capture the complex and poorly understood pathophysiology of these conditions^3^. More recently, human-based complex in vitro models (CIVMs) have emerged as promising tools to investigate rare disease mechanisms and assess therapeutic efficacy in a physiologically relevant context^4^. However, CIVMs are currently limited in scalability and integration across the full research and development pipeline, and there is a need to combine CIVMs with other human-relevant approaches to improve accuracy and efficiency in research and drug development.

While CIVMs can provide physiologically relevant readouts, their integration with in silico models remains limited by three gaps: (i) semantic interoperability, i.e., the lack of shared ontologies and metadata linking CIVM outputs (e.g., imaging, secretome, electrophysiology) to computational model variables; (ii) calibration/validation workflows, where quantitative CIVM measurements are not routinely used to calibrate mechanistic or hybrid models, nor are model predictions prospectively tested in CIVMs; and (iii) throughput and standardisation, as many CIVM protocols remain bespoke and challenging to replicate across labs. We outline a possible closed-loop workflow in which CIVMs generate standardised, annotated datasets adhering to the Findable, Accessible, Interoperable, and Reusable (FAIR) principles that parameterise or challenge digital twins/quantitative systems pharmacology (QSP)agent-based models; and model predictions then nominate the next CIVM experiment (perturbation, dose, timing). This bidirectional design could make better use of scarce patient-derived materials, may help reduce exploratory animal use, and could contribute to traceable evidence chains suitable for regulatory review. We return to this integration agenda in Section 4 with concrete steps and a prioritisation roadmap.

In this context, in silico technologies are gaining prominence as powerful tools for rare disease research. These approaches, including mechanistic models, machine learning (ML), and digital twins, can generate insights from limited datasets, integrate heterogeneous information, and simulate biological processes across scales. Their potential spans several key contexts of use (CoUs): (1) improving diagnosis and molecular characterisation through genomic and bioinformatic tools^5,6^; (2) accelerating drug discovery via virtual screening and repurposing strategies^7^; (3) supporting non-clinical development by predicting disease mechanisms and drug-target interactions^8^; and (4) informing clinical trial design using simulation-based models and digital patient cohorts^9^.

This review provides a critical synthesis of current in silico approaches in rare disease research, organised by CoUs. Rather than presenting an exhaustive list of tools, we focus on representative examples, methodological appraisal, and translational relevance. We also discuss cross-cutting challenges such as data quality, model validation, and regulatory alignment. Finally, we offer some recommendations and priority actions to enhance the integration and impact of computational technologies in addressing unmet needs in rare disease modeling and therapeutic development.

## In silico technologies across contexts of use in rare disease research

**Fig. 1:**
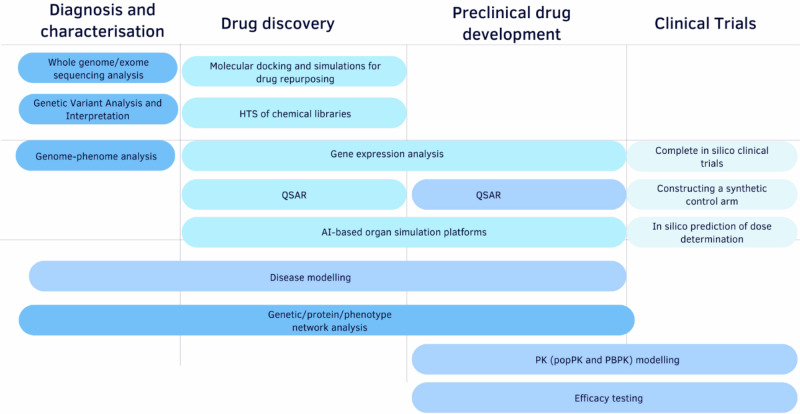
Schematic representation of in silico methods across the rare disease therapeutic pipeline. Methods span from diagnosis (e.g., variant analysis) to drug discovery (e.g., virtual screening), preclinical development (e.g., disease modeling), and clinical trials (e.g., virtual patients, pharmacokinetics/ pharmacodynamics (PK/PD) modeling). Abbreviations: PBPK physiologically based pharmacokinetics, popPK population pharmacokinetics, HTS, high-throughput screening, QSAR quantitative structure–activity relationship.

Computational tools are increasingly being deployed across the rare disease research and development continuum. As illustrated in Fig. 1, in silico methods span key contexts of use (CoUs) including: (1) diagnosis and disease characterisation, (2) drug discovery, (3) non-clinical development, and (4) clinical trial design. These approaches offer scalable, hypothesis-driven alternatives or complements to traditional in vitro and in vivo methods, especially where patient data are scarce or experimental studies are impractical.

### Diagnosis and characterisation (CoU1)

In silico tools are transforming rare disease diagnostics by integrating heterogeneous datasets and enabling earlier, more precise intervention. AI-enhanced pipelines now leverage whole-genome and exome sequencing, phenotype-extraction from electronic health records (EHRs), and deep learning for variant pathogenicity prediction^5,6^.

For instance, a recent study using natural language processing (NLP)-enhanced EHR analysis outperformed human experts in differential diagnosis of rare diseases, demonstrating higher precision and scalability^10^. However, these methods are not without limitations. Deep learning models often struggle with variants of uncertain significance, and predictive performance may not align with expert consensus, emphasising the need for hybrid human-in-the-loop pipelines^11^.

In addition, computational studies using Orphanet data suggest that borderline-common disorders involve more complex genetic architectures than ultra-rare diseases, underscoring the value of integrative genome-phenome modeling^12^. In this context, in silico tools could fill diagnostic gaps by simulating disease mechanisms where experimental models are unfeasible.

### Drug discovery (CoU2)

In silico methods play a pivotal role in accelerating and de-risking drug discovery, especially for repurposing existing compounds. Advanced platforms integrate omics data, literature mining, and network-based algorithms to identify novel therapeutic targets^7,13^. Computational docking and high-throughput virtual screening of chemical libraries enable the exploration of protein–ligand interactions at scale. Approaches like quantitative structure–activity relationship (QSAR) modeling provide rapid prioritisation of candidate molecules, although predictive reliability varies based on input data quality and chemical space coverage^14^. Importantly, AI-based drug discovery tools are increasingly shifting away from single-target paradigms toward systems-level modeling of drug–gene–phenotype interactions, enhancing their relevance for rare diseases with poorly characterised pathophysiology.

### Preclinical drug development (CoU3)

Preclinical development benefits from in silico models that simulate disease mechanisms, predict drug responses, and identify biomarkers. Platforms integrating organoids with machine learning simulations exemplify how hybrid biological-digital models can reveal mechanisms in developmental disorders^15,16^. Mechanistic multiscale models have been used to simulate the biophysical behavior of muscle tissue, offering insight into disease progression and therapeutic effects in conditions like Duchenne muscular dystrophy^17^. Endocrine organ-on-chip simulations and QSPmodels further link molecular perturbations to functional outcomes, guiding target validation and compound selection^18,19^. These tools can also inform first-in-human trial design by simulating pharmacodynamics in silico, thus accelerating development timelines and contributing to reduce animal use^20^.

### Clinical trials (CoU4)

Rare disease trials face fundamental challenges: small patient populations, ethical constraints on placebo use (especially in pediatric cohorts), and lack of standard treatments. In silico approaches such as virtual trials, synthetic control arms, and dose simulation models address these limitations^21,22^. Pharmacokinetic models are increasingly being used to extrapolate dosing, assess drug-drug interactions, and simulate pharmacodynamics across age groups and formulations^23–26^.

Together, these pharmacometric strategies support model-informed drug development, facilitating regulatory submissions and optimizing trial designs, especially where empirical approaches are infeasible^27^.

### Scope and boundaries of CoUs

CoU1 (Diagnosis/Characterisation) focuses on variant interpretation, phenotype mining, and disease stratification. CoU2 (Drug Discovery) covers target ID/prioritisation, virtual screening, and repurposing. CoU3 (Preclinical Development) addresses mechanism modeling, biomarker nomination, and in vitro/in silico efficacy prediction. CoU4 (Clinical Trial Design) includes PK/PD, PBPK, virtual cohorts, and synthetic/external controls. In real projects these CoUs are non-disjoint. For example, a splicing prediction that classifies variants (CoU1) may nominate a target (CoU2), whose network model and organoid assays calibrate a QSP model (CoU3) that informs dose selection (CoU4). We explicitly acknowledge these designed overlaps and use the CoUs as didactic lenses rather than hard boundaries. Cross-references are added where examples naturally span multiple CoUs.

## Critical synthesis of applications

### Diagnosis and characterisation (CoU1)

In silico tools for rare disease diagnosis integrate genomic sequencing, structural modeling, and machine learning to address data scarcity and phenotypic heterogeneity. Several rare diseases provide representative examples of the breadth and depth of these approaches.

Gaucher disease has been extensively studied using computational tools^28,29^. Classical methods like PCR-RFLP have been augmented with in silico tools such as SNPs3D, SIFT, PolyPhen, and I-TASSER, which predict the functional impact of novel Glucosylceramidase Beta 1 (*GBA1*) gene mutations and reconstruct mutant protein structures^30,31^. These tools offer critical insights, especially in scenarios where patient samples are scarce, by leveraging structural templates to model disease mechanisms. While they may not yet fully capture the complexity of cellular context, they represent a powerful and scalable complement to experimental approaches.

In Sandhoff disease, structure-based approaches like SWISS-MODEL, COTH, and Mutation Taster have similarly been used to assess the structural consequences of Hexosaminidase Subunit Beta (*HEXB*) mutations. Homology modeling and ligand docking have elucidated how specific variants impair enzymatic function^32–34^. While capturing complex multi-protein interactions, particularly relevant in lysosomal storage disorders, remains challenging, ongoing advances are steadily expanding the capabilities of these approaches.

More recently, deep-learning-based classifiers have been tested on diseases like cystic fibrosis and inherited retinal dystrophies using variant effect predictors such as MutPred, SpliceAI, and REVEL^35–37^. These models offer scalability and superior predictive accuracy, and ongoing efforts are addressing challenges such as ‘black box’ opacity and performance bias on ultra-rare variants to further enhance their reliability and transparency.

Comparatively, network-based approaches (e.g., Phenolyzer, STRING, Cytoscape) have been used for Ehlers-Danlos syndrome and Multiple Sclerosis to infer genotype–phenotype correlations and predict disease progression^38–40^. Their strength lies in leveraging prior knowledge, but they are sensitive to database completeness and annotation biases.

We briefly contrast three widely used tools that appear throughout rare-disease pipelines:

For ultra-rare or founder variants, it may be effective to adopt a triangulation strategy: combine REVEL (baseline missense risk) with MutPred (mechanistic hypothesis) and SpliceAI (to exclude splicing confounding). This would allow reporting of score versions/thresholds, calibration against disease-specific truth sets, and should include use of human-in-the-loop adjudication for borderline calls.

### Drug discovery (CoU2)

In silico drug discovery for rare diseases increasingly leverages AI, network pharmacology, and molecular simulation to identify new targets and repurpose existing compounds, especially where wet-lab screens are unfeasible.

Amyotrophic lateral sclerosis exemplifies AI-led drug target identification. PandaOmics, an omics-integrated AI platform, has generated novel target hypotheses by analysing transcriptomic and patient-derived datasets^41,42^. While such tools accelerate discovery and hypothesis generation, their reliance on training data can limit generalisability across diseases with sparse datasets.

For Duchenne muscular dystrophy and spinal muscular atrophy, multiomics meta-analysis combined with functional enrichment and pathway analysis tools have been used to identify shared or unique molecular pathways^43,44^. These approaches excel in revealing convergent disease mechanisms, and their effectiveness is expected to improve with advancements in sample curation and normalisation procedures.

Classical ligand docking simulations, such as those used in Ataxia Telangiectasia, enable the prediction of drug binding to Ataxia-Telangiectasia Mutated (ATM) protein targets. Studies using HADDOCK, AutoDock Vina, and DynaMut2 support virtual screening pipelines^45,46^. However, their effectiveness is limited by static structural models and assumptions about binding site accessibility.

Network pharmacology approaches, e.g., integrating gene expression signatures, protein–protein interaction maps, and drug-induced transcriptomes, have been deployed in multiple sclerosis and spinal muscular atrophy/amyotrophic lateral sclerosis research to propose repurposing candidates^44,47^. Their strength lies in context-awareness, and progressive improvements in interaction databases and platform harmonization are poised to enhance their completeness and reproducibility.

### Preclinical drug development (CoU3)

In silico models are increasingly applied in preclinical development to simulate disease pathophysiology, predict therapeutic efficacy, and identify biomarkers. These tools are particularly valuable for rare diseases, where preclinical animal models are unavailable or prove uninformative or unfeasible. A notable case is Fabry disease, where a systems biology approach combining multi-omic data and pathway modeling has been developed to identify novel biomarkers and simulate disease progression. This enabled quantitative evaluation of enzyme replacement therapies and revealed sex-specific biomarker responses^15^. As more comprehensive omics datasets become available, model applicability is expected to improve further.

Multiscale modeling platforms like the MUscle SImulation COde (MUSICO), applied in Duchenne muscular dystrophy, simulate muscle function by linking molecular interactions to tissue-level dynamics^17^. These models enable the prediction of muscle force deficits and treatment response to corticosteroids, offering rich mechanistic insights. As computational power advances and calibration techniques evolve, their accessibility and efficiency are likely to improve.

NEUBOrg, an induced pluripotent stem cells (iPSC)-derived brain organoid platform for simulating neurodevelopmental disorders, exemplifies the integration of organoid-based data with deep learning frameworks^16^. By modeling organ-level development, it supports preclinical hypothesis testing without animal models. Yet, such approaches still face some challenges in validating organoid readouts against clinical endpoints.

Another powerful approach is QSP, which integrates drug action with disease networks to simulate dose-response and predict efficacy. For example, QSP has been used in endocrine rare diseases to optimise dosing schedules and predict treatment adaptation^18,19^. Its strength lies in mechanistic fidelity, but success depends on the availability of qualified and reproducible kinetic and pharmacodynamic parameters.

### Clinical trial design (CoU4)

Rare disease clinical trials are challenged by small sample sizes, lack of comparators, and ethical concerns, particularly in pediatric populations. In silico technologies can mitigate these issues by simulating virtual populations, optimising dose regimens, and replacing or augmenting trial arms.

Virtual clinical trials have been applied in diseases like congenital pseudarthrosis of the tibia, where patient recruitment is extremely limited^22^. Using digital twin simulations, researchers tested dose–response relationships and predicted efficacy in pediatric subgroups, reducing the reliance on placebo-controlled arms. However, the accuracy of these models depends on the fidelity of the input clinical data.

Physiologically based pharmacokinetic (PBPK) modeling is being widely used to simulate absorption, distribution, metabolism, and excretion (ADME) in diverse populations. For example, PBPK models have been applied in rare metabolic diseases to extrapolate dosing for neonates and to predict drug–drug interactions^23–25^. These models offer valuable mechanistic transparency, but require extensive physicochemical and anatomical parameters, which are often unavailable for rare cohorts.

Population pharmacokinetic (popPK) and PK/PD (pharmacokinetic/pharmacodynamic) models adopt a top-down approach, leveraging clinical data to predict exposure–response relationships^26^. These are particularly useful when trial data are limited, and with careful qualification procedures, the risk of overfitting could be effectively minimised. In rare diseases, they often support regulatory decisions regarding dosing.

Emerging practices also include the construction of synthetic control arms, where in silico cohorts are generated from historical or real-world data to substitute for placebo groups. This has been piloted in several rare neurodegenerative disorders and external control data are beginning to be accepted by regulators, most frequently for drugs for rare diseases^21^.

## Challenges and future priority actions

Despite substantial progress, the application of in silico technologies to rare disease research continues to face systemic challenges that limit scalability, reproducibility, and translational impact. These challenges are not merely technical but also relate to data governance, model validation, regulatory alignment, and ecosystem readiness. Addressing them will likely require sustained, coordinated effort across disciplines and stakeholders. Rare diseases inherently suffer from limited patient numbers and fragmented data sources. Many computational pipelines, especially those relying on machine learning, require large, diverse, and well-annotated datasets to achieve robust performance. Yet, in rare disease contexts, data may be siloed, incomplete, or non-standardised across institutions or registries. Efforts such as GA4GH (https://www.ga4gh.org/), IRDiRC (https://irdirc.org/), and FAIR (Findable, Accessible, Interoperable, and Reusable) data initiatives aim to promote interoperable and reusable data formats, but uptake remains inconsistent. Even when genomic or clinical data are available, phenotypic granularity (e.g., detailed longitudinal clinical annotations) is often lacking. This particularly undermines the performance of deep learning models and limits external validation. Investment in federated data infrastructures, harmonised annotation standards (e.g., HPO (https://hpo.jax.org/), OMOP (https://ohdsi.github.io/CommonDataModel/)), and incentives for data sharing in compliance with privacy regulations will be critical to scale computational approaches responsibly.

To move beyond declarative FAIR claims, we apply an indicator-based self-assessment covering persistent identifiers (PIDs) - such as such as digital object identifiers (DOIs) -, machine-readable metadata, vocabulary alignment, access/authorisation, licensing, provenance, versioning, and reuse evidence. We report these elements concisely in Box 1 and reference them in the Data/Code Availability statements for each asset cited in this work.

Regarding benchmarking transparency, we provide a concise, container-first description of the computational environment and exact artifacts needed for end-to-end reproduction. Box 2 lists the items we require for all benchmarks in this paper (and future releases): container image with digest, dependency lockfiles, deterministic seeds, dataset PIDs/checksums, frozen splits, hardware notes, and a one-command runner to regenerate figures/tables.

It is worth mentioning that this review did not generate new datasets or code; Boxes 1 and 2 outline reporting standards we recommend for future releases.

In silico methods often lack standardised validation protocols, which complicates their interpretation and acceptance. While some tools undergo retrospective benchmarking using public datasets, prospective or experimental validation is rarely conducted. This is especially problematic for models with regulatory implications (e.g., PBPK or digital twins in clinical trials). Moreover, reproducibility is hampered by poor documentation of model assumptions, lack of code availability, and versioning issues in ML frameworks. Mechanistic models, while often more interpretable, may suffer from overfitting due to the high number of parameters relative to available data. Adoption of formal model credibility frameworks (e.g., Verification, Validation, and Uncertainty Quantification (VVUQ)), pre-registration of modeling protocols, and incentives for open-source model sharing are urgently needed (Table 1).

**Table 1:** Comparative considerations for variant effect predictors used in CoU1 pipelines (MutPred, SpliceAI, REVEL), summarising focus, methods, strengths/limitations, and practical usage tips

Tool	Primary focus	Method sketch	Typical strengths	Typical limitations	Practical tip
MutPred	Missense pathogenicity & mechanism hypotheses	Ensemble/ML over protein sequence/structure features	Proposes putative mechanisms (e.g., loss of PTM site); useful for hypothesis generation	Depends on feature availability/quality; less informative for non-missense variants	Use when you want mechanistic hints to design follow-up assays
SpliceAI	Splice impact (donor/acceptor gain/loss)	Deep neural network on genomic context	State-of-the-art for splice-altering variants; captures long-range sequence signals	Not applicable to most purely missense effects; can overcall borderline scores without RNA evidence	Pair with RNA-seq or minigene assays where feasible
REVEL	Missense pathogenicity meta-score	Ensemble aggregator of multiple predictors	Robust baseline ranker across genes; easy triage	Black-box ensemble; gene/constraint context not explicit	Use as a ranker, then layer gene-/disease-specific priors and expert curation

A key barrier to clinical adoption lies in the fragmentation between in silico development and experimental or regulatory pathways. Computational models are often used in early discovery, but can fail to influence downstream decisions in preclinical or clinical development due to siloed workflows or lack of interoperability with lab or trial systems. This disconnect is particularly visible in drug repurposing efforts, where computational predictions are rarely followed by systematic validation in either in vitro or animal models. Similarly, few digital clinical trial simulations are integrated into actual trial protocols submitted to regulatory bodies. Establishing closed-loop workflows, where in silico insights feed into experimental designs, and vice versa, can enhance reliability and speed. Embedding computational scientists into translational teams and adopting common Application Programming Interfaces across platforms will support integration.

Regulatory guidance on the use of in silico tools in rare disease contexts remains limited, fragmented across therapeutic areas, and largely reactive. While there is growing openness to model-informed drug development, most agencies still evaluate computational models on a case-by-case basis. This limits their scalability and deters industry adoption. Notably, digital evidence frameworks are still evolving, and the qualification of in silico tools as drug development tools under the European Medicines Agency (EMA) or the US Food and Drug Administration (FDA) pathways remains a time-consuming process with unclear benefit/risk trade-offs for sponsors working in rare disease. Co-development of regulatory sandboxes, model qualification pathways, and shared validation datasets for rare diseases could help lower barriers to adoption. Multi-stakeholder consortia should include regulators from the outset of model development.

Finally, in silico approaches risk amplifying existing inequities, if not designed and validated with attention to underrepresented populations. Genomic reference datasets are still skewed toward individuals of European ancestry. Bias may also arise in the simulation of clinical trials. For example, a study demonstrated that patients with rare diseases in India are seldom represented in clinical trials^48^. Consequently, datasets used for in silico modelling in clinical trial design (CoU4) may lack adequate representation. In addition, predictive algorithms may inadvertently perform worse on rare diseases prevalent in underrepresented ethnic groups or geographies. Additionally, lack of interpretability in AI models may erode clinician and patient trust, especially where high-stakes decisions are being made (e.g., diagnosis, trial eligibility). Building trust requires transparency, explainability, and participatory model design. Diversification of training datasets, inclusion of community perspectives, and development of explainable AI frameworks tailored to rare diseases are essential for equitable deployment.

As rare disease research continues to embrace computational methods, the field stands at an inflection point. Technical innovation alone is not sufficient. Addressing structural, methodological, and regulatory gaps is essential to ensure these tools are not only powerful in theory but impactful in practice. The next decade offers a unique window to institutionalise in silico technologies as a core pillar of rare disease translational science, provided we act with urgency, coordination, and transparency.

A set of priority actions to advance the integration and regulatory approval of innovative in silico technologies for rare disease modeling, drug discovery, and clinical trial design are reported in Table 2.

**Table 2:** Possible priority actions to support the integration and regulatory acceptance of in silico technologies for rare disease research

Category	Priority Action	Responsible interest-holders	Priority (timeline)	Feasibility	Key dependencies between actions / notes
Data Infrastructure & Standards	1. Develop standardised protocols for data generation, annotation, and sharing	Research Institutions, Data Consortia, Regulatory Agencies, Funding Bodies	Short-to-mid	Medium	Depends on #8; aligns to HPO/OMOP; feeds #5.
Data Infrastructure & Standards	2. Establish centralised, high-quality repositories for rare disease data	Governments, Research Institutions, Data Governance Bodies, Funding Agencies, Regulatory Agencies	Mid-to-long	Medium	Funding and governance heavy; leverages federated designs.
Data Infrastructure & Standards	3. Ensure diversity and inclusivity in data repositories	Research Institutions, Data Consortia, Policymakers, Patient Advocacy Groups, Regulatory Agencies, Ethicists	Mid-term	Medium	Depends on #1, #2, #8 and #11; requires targeted recruitment.
Model Development, Validation & Regulation	4. Enhance interdisciplinary collaboration for advanced computational modeling	Research Institutions, Universities, Industry, Funding Agencies	Short-term	High	Low cost; accelerates all other actions.
Model Development, Validation & Regulation	5. Develop validation frameworks for computational models to enable regulatory acceptance	Regulatory Agencies, Standards Organisations, Research Consortia	Mid-term	Medium	Builds on existing VVUQ; prerequisite for #6 and #13.
Model Development, Validation & Regulation	6. Harmonise global regulatory standards for in silico technologies	Regulatory Agencies, International Regulatory Collaborations	Long-term	Low-to-Medium	Requires #5 and cross-agency fora.
Data Infrastructure & Standards	7. Implement ethical guidelines for synthetic data use, privacy, and consent	Ethics Committees, Research Institutions, Policymakers, Regulatory Bodies	Short-to-mid	High	Needs patient-group input; underpins #2, #3 and #9.
Governance, Ethics & Patient Involvement					
Data Infrastructure & Standards	8. Promote open-access principles and equitable data sharing	Research Institutions, Governments, International Organisations	Short-term	High	Works immediately via policy updates; enables #1, #2, and #3.
Data Infrastructure & Standards	9. Leverage blockchain for secure and transparent data sharing	Tech Industry, Data Governance Bodies, Research Institutions	Long-term / exploratory	Low	Considers pilots after #2 and #7; should account for cost/benefit.
Capacity Building & Awareness	10. Develop targeted education and training programs on in silico tools	Academic Institutions, Industry, Professional Societies, Regulatory Agencies	Short-term	High	Requires alignment with regulators/clinicians.
Governance, Ethics & Patient Involvement	11. Increase advocacy efforts with policymakers and patient organisations	Patient Advocacy Groups, Policymakers, Research Institutions	Mid-term	Medium	Catalyses funding/standards for #2 and #6.
Capacity Building & Awareness					
Governance, Ethics & Patient Involvement	12. Engage patients as co-creators in research design	Research Institutions, Patient Organisations, Funding Bodies	Mid-term	Medium	Co-creates governance for #7, improves adoption.
Model Development, Validation & Regulation	13. Foster investment in digital twins and AI-driven rare disease modeling	Governments, Industry, Research Institutions, EU Initiatives (e.g., Virtual Human Twins)	Long-term	Medium	De-risked by #5 and #6.

The table includes responsible interest-holders, timelines, feasibility, and key dependencies between actions. Actions are grouped into four main categories: (i) Data Infrastructure & Standards, (ii) Model Development, Validation & Regulation, (iii) Governance, Ethics & Patient Involvement, and (iv) Capacity Building & Awareness.

Timelines:

- Short-term (0–12 months): Immediate actions that can be planned and executed within a year.

- Medium-term (12–24 months): Actions requiring more preparation, resource allocation, or coordination, achievable within two years.

- Long-term (24–36 months): Strategic actions that need sustained effort, policy shifts, or broader interest-holder alignment, achievable within three years.

- Exploratory ( ≥ 36 months): Forward-looking initiatives that are high-impact but uncertain, dependent on major innovation, regulatory changes, or long-term investment.

Feasibility:

- Low: Actions face major barriers (e.g., limited evidence, fragmented infrastructure, low awareness, or no supportive policy/regulatory framework), making near-term implementation unlikely.

- Medium: Actions are supported by emerging evidence, partial infrastructure, growing interest-holder interest, or pilot initiatives, but require significant coordination and investment to scale.

- High: Actions build on established practices, available infrastructure, broad interest-holder support, and existing regulatory/policy frameworks, making them readily implementable.

Box 1 FAIR maturity mini-checklist (report once per dataset/model/code asset)

- PID/DOI: global, resolvable identifier provided.
- Metadata (machine-readable): e.g., DataCite/schema.org/RO-Crate, minimally complete.
- Vocabularies: controlled terms (e.g., HPO/OMIM/ORDO; OMOP where applicable).
- Access protocol (A1): standard protocol (HTTPS/GA4GH DRS), with uptime policy.
- AuthN/Z (if controlled): method stated (e.g., OAuth, DAC), and rationale.
- License: explicit, human- and machine-readable (e.g., CC BY 4.0 / Apache-2.0).
- Provenance: workflow/configs and who/when (e.g., PROV-O, RO-Crate).
- Versioning: semantic version/release tag and changelog.
- Reuse evidence: citations/downloads or statement of first release.

Box 2 Reproducible environment checklist

- Container image (Docker/Singularity) + immutable digest.
- Lockfiles/manifests for dependencies (e.g., requirements.txt, renv.lock, environment.yml).
- Determinism controls: fixed random seeds; note any nondeterministic ops.
- Data identifiers: dataset PIDs/URLs + checksums; date of retrieval.
- Splits: frozen train/val/test splits or cross-val protocol committed to repo.
- Hardware/accelerators: CPU/GPU model and key runtime flags.
- Runner: single command/script (make repro) that rebuilds results/figures.
- Signed artifacts (optional): attestations/checksums of final outputs.

### Ethical risks and mitigations in rare-disease in silico modelling

Rare-disease datasets are small and identifiable; digital twins and related models can therefore amplify both benefit and harm. Below, we offer some suggestions for concrete risks and how we mitigate them:

- Re-identification via linkage or model inversion
  - *Mitigation:* Data minimisation; controlled access; privacy-preserving analytics (federation; where applicable, differential-privacy bounds disclosed); k-anonymity for shared metadata; an explicit residual-risk statement in Data/Code Availability.
- Misuse of digital twins for exclusionary decisions (e.g., coverage, employment)
  - *Mitigation:* Purpose limitation and usage contracts; clear model cards stating intended use, limitations, and prohibited use; access auditability; disclaimers against non-clinical decision-making.
- Automation bias and unsafe propagation of repurposing suggestions
  - *Mitigation:* Evidence labels and uncertainty ranges on outputs; predefined decision thresholds; human-in-the-loop review; preclinical validation gates before any clinical communication; guardrails that suppress paediatric dosing or contraindicated recommendations without expert sign-off.
- Bias and inequity across ancestry, sex, and age
  - *Mitigation:* Stratified performance reporting; minimum performance floors before deployment; bias diagnostics with corrective reweighting/retraining; targeted data collection to close gaps; clear statement when a model is not fit for a subgroup.
- Paediatrics and other vulnerable populations
  - *Mitigation:* Separate validation/calibration for paediatric/vulnerable cohorts; conservative decision policies; explicit contraindications when validation is insufficient; strengthened consent/assent language.
- Psychological harm from prognostic simulations
  - *Mitigation:* Communicate ranges and uncertainty; present scenarios as decision support, not destiny; ensure clinician-mediated interpretation and provide patient opt-out.

## Conclusions

In silico technologies are increasingly influencing the landscape of rare disease research, offering new avenues to address long-standing challenges in diagnosis, drug discovery, preclinical evaluation, and clinical trial design. Their capacity to integrate heterogeneous data, simulate biological complexity, and generate actionable predictions makes them particularly well-suited to the unique constraints of rare diseases, namely, limited patient populations, fragmented data, and high phenotypic variability. Yet the promise of these tools must be tempered by a critical understanding of their current limitations and evolution of possible strategies to minimise or overcome these barriers. Issues of data quality, validation standards, interpretability, and regulatory alignment continue to restrict their broader adoption and clinical impact. Across all contexts of use, the most promising advances are emerging not from standalone tools, but from integrated, hybrid approaches that combine computational predictions with experimental feedback and expert oversight.

To realise their full potential, in silico methods should be embedded into the translational pipeline as trusted, interoperable components, supported by open data practices, validated modeling frameworks, and inclusive development strategies. This will require not only methodological innovation but also ecosystem-wide coordination among researchers, clinicians, regulators, and patient communities.

Ultimately, the value of in silico technologies shall be measured not by their technical sophistication alone, but by their ability to accelerate meaningful progress for individuals affected by rare diseases. With deliberate investment and sustained collaboration, they are poised to become a cornerstone of precision translational research in this high-need domain.

This review is narrative and non-systematic, relying on expert selection of examples rather than a formal systematic search. As a result, it may be subject to selection bias and cannot claim to be exhaustive of all in silico technologies or disease areas. The evidence base on which we draw is itself uneven: many of the computational approaches highlighted are still experimental, published only in proof-of-concept form, or evaluated retrospectively on small datasets. Prospective validation, particularly in rare-disease contexts, remains limited and is rarely benchmarked across independent groups. Furthermore, most of the literature we review comes from English-language sources and from regions with well-resourced research infrastructures, which may underrepresent work carried out in low- and middle-income countries or in non-English publications. We also acknowledge that rare-disease patient populations are highly heterogeneous and often underrepresented in public datasets, which constrains the generalisability of computational models and risks amplifying existing biases. Finally, because our focus is on methodological trends and translational opportunities rather than on regulatory submissions per se, some nuances regarding jurisdiction-specific regulatory guidance could not be fully explored.

## Data Availability

Data sharing is not applicable to this article as no datasets were generated or analysed during the current study.
